# *EWSR1* Rearrangement and CD99 Expression as Diagnostic Biomarkers for Ewing/PNET Sarcomas in a Moroccan Population

**DOI:** 10.1155/2018/7971019

**Published:** 2018-09-18

**Authors:** Sara Louati, Nadia Senhaji, Laila Chbani, Sanae Bennis

**Affiliations:** ^1^Bioactive Molecules, Structure and Functions Laboratory, Faculty of Sciences and Techniques, Sidi Mohamed ben Abdellah University of Fez, Morocco; ^2^Pathological Anatomy and Molecular Pathology Department, Hassan II University Hospital of Fez, Morocco; ^3^Biomedical and Translational Research Laboratory, Faculty of Medicine and Pharmacy, Sidi Mohamed ben Abdellah University of Fez, Morocco

## Abstract

Ewing sarcoma/primitive neuroectodermal tumor (Ewing/PNET sarcomas or EPS) are a group of round cell tumors. Malignant round cell tumors form a large and diverse group that includes rhabdomyosarcoma, synovial sarcoma, non-Hodgkin's lymphoma, neuroblastoma, hepatoblastoma, Wilm's tumor, desmoplastic small round cell tumor, and other morphologically similar entities. Differential diagnosis of Ewing sarcoma/primitive neuroectodermal tumor (Ewing/PNET sarcomas or EPS) is difficult. In addition to morphology and immunohistochemistry (IHC), differential diagnosis of these tumors is based on molecular analysis of the *EWSR1* gene rearrangement using fluorescent in situ hybridization (FISH) technique. We investigated the diagnostic value of combined CD99 immunostaining and *EWSR1* t(22q12) alteration using a dual-color, break-apart rearrangement probe in forty-one formalin-fixed paraffin-embedded (FFPE) tissue samples from pediatric and adult patients diagnosed with EPS. IHC was performed in all cases using the CD99 antibody and showed a positivity of 92.7% in the enrolled cases (38/41) followed by FISH analysis where 48.8% of the cases (20/41) were rearranged. Sensitivity and specificity for IHC assays were 88% and 58%, respectively. Notably, FISH had a sensitivity of 100% and a specificity of 87%. In addition, CD99 positivity was found to correlate with *EWSR1* rearrangement (*p* < 0.05). This report shows that FISH has better sensitivity and specificity than IHC in the Moroccan population, and supports its combination with CD99 immunostaining as diagnostic biomarkers for this rare malignant entity.”

## 1. Background

Small round cell tumors are highly aggressive malignant tumors that are characterized by small and relatively monotonous, undifferentiated cells. This group consists of Ewing sarcoma, PNETs, rhabdomyosarcoma, synovial sarcoma, non-Hodgkin's lymphoma, retinoblastoma, neuroblastoma, hepatoblastoma, nephroblastoma, small cell osteogenic sarcoma, Wilm's tumor, and desmoplastic small round cell tumor. The undifferentiated or poorly differentiated primitive character of the tumor cells, as well as the rare occurrence of these tumors relative to the more differentiated and common carcinomas, makes differential diagnosis of small round cell tumor types particularly difficult. Ewing sarcoma (OMIM: 612219) was first described in 1921 by the American pathologist James Stephen Ewing (named as diffuse endothelioma of bone) [1]. This rare and aggressive tumor is characterized by small round cells that occur frequently in soft tissue and bone of adolescents and young adults [2]. Subsequently, it was noted that some of these tumors show features of neural differentiation (pseudorosettes of Homer–Wright and positive Periodic acid–Schiff (PAS) stain), which suggested the presence of a new histological subentity [2, 3]. Tumor variants with these neural features have been named Ewing/PNET sarcomas (EPS). The identification of this entity in routine histopathological examinations has improved significantly with the emergence of IHC and molecular biology techniques. Most of EPS tumors express CD99, which is a highly sensitive immunohistochemical biomarker [4]. However, this marker lacks specificity and can be positive in other sarcomas and lymphomas [4]. In addition, CD57, synaptophysin, chromogranin, vimentin, neuron-specific enolase (NSE), and S-100 are often expressed in EPS tumors [2, 4]. Importantly, these tumors show frequent and specific rearrangement of the Ewing sarcoma breakpoint region 1 (*EWSR1*) gene located on chromosome 22 [5, 6]. Translocations between *EWSR1* and Friend leukemia integration 1 transcription factor (*FLI1*) (85%, t(11;22)(q24;q12)) and between *EWSR1* and ETS transcription factor (*ERG*) (10%, t(21;22)(q22;q12)) are key differential cytogenetic alterations in EPS [7]. These chimeric fusions encode for multifunctional proteins encompassing various cancer hallmarks such as evading growth suppressors, sustaining proliferative signaling, resisting cell death, inducing angiogenesis, and activating invasion and metastasis which are believed to drive the tumorigenesis of this rare entity [5]. Specific FISH with break-apart probe targeting translocations on FFPE sections is commonly used in combination with CD99 immunostaining for diagnosis of these tumors.

Morocco harbors a unique population that is a complex admixture of autochthonous Maghrebi (including Berber-speaking groups) and European, Northwest African, West African, and West Asian lineages, with various degrees of intermixing. Here, we report the results of a study evaluating the sensitivity and specificity of FISH-based *EWSR1* rearrangement, as well its correlation with CD99 positivity, for enhanced diagnosis of EPS in a large Moroccan cohort.

## 2. Materials and Methods

### 2.1. Patient Selection and Data Collection

The study protocol was approved by the ethics committee of Hassan II University Hospital of Fez, Morocco. FFPE tissue from cases coded as EPS was retrieved from the Pathology Department of Hassan II University Hospital of Fez, Morocco. All cases were histologically reviewed and the diagnosis of EPS was based on histology and IHC according to the latest World Health Organization (WHO) classification of tumors of soft tissue and bone [8]. Cases with insufficient or poor quality of tissue were excluded. A total of forty-one cases of pediatric and adult patients diagnosed with EPS were included in this study. In addition, cases with round cell liposarcomas (*n* = 5), desmoplastic small round cell tumors (*n* = 2), and embryonic rhabdomyosarcomas (*n* = 8) were included as comparative controls.

### 2.2. Immunohistochemical Analysis

IHC analysis was performed in all cases (41 cases of EPS and 15 controls). Sections (5 μm thick) were prepared from FFPE tissue blocks and stained with hematoxylin, eosin, and safranin (HES). Immunostaining was performed with CD99 Rabbit Monoclonal Antibody (EPR3097Y, dilution: 1 : 100, Cell Marque™, Sigma-Aldrich®) using the LSAB kit (DakoPatts®, Copenhagen, Denmark) with an automated immunostainer. Pretreatment using a heat-induced epitope retrieval (HIER) procedure prior to IHC was performed and DAB (3,3′-diaminobenzidine tetrahydrochloride) was used as a chromogen. Appropriate positive and negative control samples were used thoroughly. Results were considered positive when >10% membranous staining was observed in tumor cells.

### 2.3. Fluorescence *In Situ* Hybridization

The FISH analysis was performed in all cases (41 EPS cases and 15 controls). Sections (3.5 μm thick) from FFPE tissue were processed for FISH using the EWSR1 (22q12) dual-color break-apart rearrangement probe (Vysis FISH, Abbott Laboratories®). The FISH was then performed according to the manufacturer's recommendation. In normal cells lacking t(22q12), a two-fused signal pattern (yellow) is expected to reflect two intact copies of the *EWSR1* gene. In abnormal cells with t(22q12), a split signal is observed (one green and one orange signal pattern). For each sample, a minimum of 100 nonoverlapping tumor cells were evaluated to assess the presence of fused or split green and red signals. A positive result was defined as >30% of cells with split signals.

### 2.4. Statistical Analysis

Sensitivities, specificities, positive predictive values (PPV), and negative predictive values (NPV) were calculated using the final pathologic diagnosis as the gold standard and defined as follows: sensitivity = true positives/(true positives + false negatives); specificity = true negatives/(true negatives + false positives); PPV = true positives/(true positives + false positives); NPV = true negatives/(true negatives + false negatives). Epi Info™ software, version 7.2, was used to perform all analyses. Youden's J statistic (Youden's Index = (sensitivity + specificity) − 1) [9] was used for diagnostic accuracy. A *χ*^2^ test was used to correlate CD99 positivity with the presence of *EWSR1* rearrangement. The results are considered significant when *p* < 0.05.

## 3. Results

### 3.1. Clinical Features

This retrospective study was conducted over 85 months (from January 2010 to January 2017). The average age of patients was 24 years, ranging from 3 to 62 years (median, 19 years) with male predominance (27 males/14 females). 29.3% of the cases (12/41) occurred in the lower limbs, 17.1% (7/41) in the thorax, 12.2% (5/41) in the abdomen, 12.2% (5/41) in the upper limbs, 12.2% (5/41) in the pelvis, and 2.4% (1/41) in the head and neck. For 14.6% (6/41) of the cases, the only information provided concerning the location was just the soft tissue. All EPS cases have been reviewed by a pathologist, diagnosed and graded III according to the FNCLCC (Fédération Nationale des Centres de Lutte Contre le Cancer) classification (Table 1).

### 3.2. Histopathology and IHC Analyses

**Figure 1:**
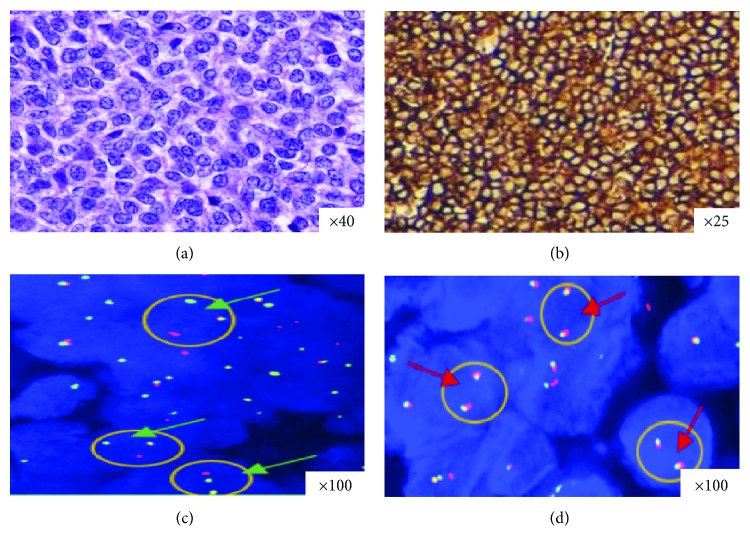
H&E staining, CD99 immunostaining, and dual-color FISH for the *EWSR1* gene in selected EPS cases. (a) H&E stained section showing diffuse proliferation of round blue cells. (b) Diffuse, intense, and membrane expression of CD99 antibody. (c) Dual-color FISH performed with break-apart EWSR1 probes reveals nuclei in which one pair of the probe signals is split apart due to a rearrangement in the *EWSR1* gene (green arrows). Red arrows indicate a two-signal pattern in normal cells (d).

EPS are composed of proliferating small round cells (Figure 1(a)). Mitotic activity ranged from 4 to 20 mitoses per 10 high power fields (median, 10 mitoses). Areas of tumor necrosis were present in 92.7% of the cases (38/41), including 24 cases displaying more than 50% and 14 cases with less than 50%. 92.7% of the cases (38/41) showed a positivity for CD99 antibody, including 29 cases with strong, diffuse, and characteristic membranous staining (Figure 1(b)). The CD99 antibody was negative in all 15 comparative controls.

### 3.3. Cytogenetic Analysis

The FISH analysis showed a rearrangement of the *EWSR1* gene locus in 48.8% of the cases (20/41) (Figure 1(c)). In 7.3% of the cases (3/41), interpretation of hybridization signals was not possible because of weak signals, insufficient tissue, or poor tissue fixation. FISH was negative for all 15 cases used as comparative controls (Figure 1(d)).

### 3.4. Sensitivity, Specificity, NPV, and PPV

Table 2 summarizes sensitivities, specificities, PPV, NPV, and Youden's index for each of CD99 antibody and FISH with the *EWSR1* (22q12) dual-color, break-apart rearrangement probe for the diagnosis of EPS. IHC of CD99 showed a positivity of 92.7% in the enrolled cases (38/41). FISH showed that 48.8% of the cases (20/41) were rearranged. Sensitivity and specificity for IHC assays were 88% and 58%, respectively. Notably, FISH had a better sensitivity (100%) and specificity (87%). In terms of PPV, the FISH technique (89%) predicts the disease better than IHC (48%). The Youden's index of *EWSR1* break-apart (0.9) was near the value of 1 which supports the FISH technique as an accurate diagnostic tool for EPS. Furthermore, we found a strong correlation between the diffuse positivity ≥ 50% of CD99 and the presence of *EWSR1* rearrangement (*p* < 0.05) (Table 3).

## 4. Discussion

Ewing sarcoma and PNET are aggressive round cell tumors and considered as two morphologic variants of the same entity (EPS) because they show similar clinical, immunohistochemical, and molecular profiles [7]. These tumors usually arise in bone and occasionally in deep soft tissues of the paraspinal region, the chest wall, and the lower extremities in children and young adults [8]. Currently, the EPS family is defined by the presence of specific translocations frequently involving the *EWSR1* gene, which is fused to an E26 transformation-specific (*ETS*) family gene (*FLI-1*, *ERG*, or ETS variant 1 (*ETV1*)) [10]. In addition, they also express the membranous CD99 which is characteristic and sensitive but not specific [11]. A combination of histopathology examination, IHC, and FISH techniques is the gold standard for the diagnosis of EPS.

We have performed a comprehensive investigation of combined CD99 immunostaining and *EWSR1* rearrangement as diagnostic biomarkers to increase their sensitivities and specificities in a large cohort of Moroccan EPS patients. In this study, we showed that combining IHC and FISH (CD99 and *EWSR1* rearrangement) is more sensitive and specific than each test alone for the diagnosis of EPS. The dual-color, break-apart FISH technique is used in several pathology laboratories to identify the rearrangement of the *EWSR1* gene with a potential added value in the diagnosis of EPS [12, 13]. Furthermore, it is considered as a standard of choice because it can be easily performed in FFPE tissues with a good preservation of tissue architecture [14]. Previously, Mhawech-Fauceglia et al. found a sensitivity of 50% in a similar study, which is significantly less than our result (100%) [14]. This difference between the two studies could be explained by the number of recruited cases that can affect the statistical analysis and the conservation of the FFPE tissue or even the duration of sample fixation. The fixation is certainly the most limiting step and the most difficult. This time setting affects the quality of genetic materials by modifying the structure of nucleic acids (RNA, DNA) as well as proteins [14]. The IHC technique showed low specificity (58%) and low positive and negative predictive values (48% and 92%, respectively) compared to the FISH technique (specificity 87%, positive predictive values 89%, and negative 100%). However, assessment of CD99 immunoreactivity was useful for the selection of EPS cases that may be candidates for molecular testing by FISH. Tumors containing <50% of CD99 positive cells had low probability to be rearranged as demonstrated by the *χ*^2^ test (*p* < 0.005). CD99 is not specific for Ewing sarcoma and can be positive in other cancers such as poorly differentiated synovial sarcomas, small round cell osteosarcomas, rhabdomyosarcomas, gliomas, and granulosa cell tumors [8]. Therefore, CD99 alone might not be used as an accurate diagnostic immunohistochemical biomarker of EPS. It was suggested that a combination of CD99 and FLI1 may increase the accuracy of IHC [15].

While the FISH break-apart method provided some clear advantages, its main disadvantage is that the translocation partner is not identified, which is important in predicting tumor behavior. As *EWSR1* rearrangement may be found in other tumors such as desmoplastic small round cell tumors [16], a detection of fusion types (*EWSR1/FLI-1* and *EWSR1/ERG*) from RNA is necessary to confirm the diagnosis using reverse transcription polymerase chain reaction (RT-PCR), which is a highly specific method [3, 17]. Prognosis of this entity also depends on several factors including the location and volume of the tumor, the stage, the age, and the response to chemotherapy [18]. Current treatment allows 5-year survival of ~70% for localized stages but less than 35% in EPS patients with advanced disease [19]. Our study showed that *EWSR1* rearrangement and CD99 expression are potential biomarkers for diagnosis of EPS, and more advanced investigation to study their correlations with prognosis, survival, and therapy response are awaited.

## 5. Conclusion

In order to correctly diagnose EPS, laboratory testing should include both histopathology and CD99 immunostaining, as well as molecular analysis using a dual-color, break-apart rearrangement probe in order to better characterize this entity by identifying the *EWSR1* rearrangement. Large prospective series to validate these results are urgently needed especially with the rareness of these highly malignant sarcomas.

## Figures and Tables

**Table 1:** Clinical features of enrolled 41 EPS cases.

Case number	Age and sex	Anatomic site	*EWSR1* gene rearrangement	CD99 staining
1	26/M	Abdomen	−	+++
2	37/M	Lower limbs	−	+
3	10/F	Head and neck	+	+++
4	40/F	Lower limbs	+	+
5	14/M	Soft tissue	−	+++
6	16/M	Upper limbs	+	−
7	19/M	Lower limbs	+	+
8	10/M	Lower limbs	−	+
9	8/F	Lower limbs	+	+++
10	19/M	Lower limbs	+	+++
11	10/M	Pelvis	−	+
12	13/M	Upper limbs	+	+++
13	38/M	Pelvis	−	+++
14	6/F	Pelvis	IN	+++
15	19/F	Upper limbs	+	+++
16	8/M	Lower limbs	+	+++
17	18/M	Abdomen	−	+++
18	5/M	Thorax	+	+++
19	9/F	Lower limbs	+	+++
20	15/M	Lower limbs	−	+++
21	22/M	Thorax	−	+++
22	11/M	Soft tissue	−	+++
23	23/F	Thorax	+	+++
24	17/M	Thorax	+	+++
25	6/F	Thorax	−	+++
26	21/M	Soft tissue	−	+++
27	18/M	Lower limbs	IN	+
28	21/F	Pelvis	+	+++
29	62/F	Abdomen	−	+
30	28/F	Soft tissue	−	+++
31	29/M	Abdomen	−	−
32	14/M	Lower limbs	+	+++
33	25/M	Upper limbs	+	+++
34	3/M	Lower limbs	+	+++
35	29/M	Pelvis	+	+++
36	28/M	Soft tissue	−	+++
37	50/F	Soft tissue	+	+++
38	40/M	Thorax	−	+
39	62/F	Upper limbs	−	+
40	37/M	Thorax	—	−
41	21/F	Abdomen	+	+++

For *EWSR1* rearrangement: positive (+), negative (−), and inconclusive (IN); for CD9 immunostaining: positive staining < 50% (+) and strong, diffuse, and membranous staining ≥ 50% (+++).

**Table 2:** Comparison between IHC and FISH test for the diagnosis of EPS.

	CD99 antibody	*EWSR1* break apart
Number of cases	56	56
Sensitivity	88%	100%
Specificity	58%	87%
PPV	48%	89%
NPV	92%	100%
Youden's index	0,55	0,9

Abbreviations: NPV: negative predictive value, PPV: positive predictive value.

**Table 3:** Association of *EWSR1* rearrangement and CD99 positivity.

	CD99 positivity						
	<50%			≥50%			*P* value
*EWSR1* rearrangement	+	—	IN	+	—	IN	0.021
Ewing sarcoma	2	0	1	17	0	2	
Non Ewing sarcoma	0	6	0	0	10	0	

IN: FISH was inconclusive.

## Data Availability

A laboratory notebook explaining all the steps is available in the laboratory. The data is scanned and stored in two external hard drives and available from the corresponding author on request.
